# A comparison between venous blood sampling and capillary volumetric absorptive microsampling for antibiotics levels monitoring in individuals with and without periodontal disease

**DOI:** 10.1007/s00784-025-06466-3

**Published:** 2025-08-23

**Authors:** Ioanna Lazaridi, Eva Choong, Thomas Mercier, Laurent A. Decosterd, Catherine Giannopoulou, Alkisti Zekeridou

**Affiliations:** 1 Division of regenerative dental medicine and periodontology, University clinics of dental medicine, University of Geneva, Geneva, Switzerland; 2 Laboratory & Service of Clinical Pharmacology, Department of Laboratory Medicine and Pathology, University Hospital of Lausanne and University of Lausanne, Lausanne, Switzerland

## Abstract

**Objectives:**

We aimed to compare the antibiotic concentrations obtained using the volumetric absorptive microsampling (VAMS) devices with those determined in plasma from conventional venous blood collected within the frame of a pharmacokinetic study of amoxicillin (AMO), metronidazole (MET), azithromycin (AZI), commonly used for periodontal treatment. The suitability and overall, acceptability of the VAMS approach was also ascertained by both participants of the pilot study and dentist practitioners.

**Materials and methods:**

Twelve volunteers (6 subjects without periodontal problems (PH), and 6 individuals affected with periodontitis (PP)) were administered 500 mg each of amoxicillin, metronidazole, and azithromycin. Paired venous blood (VB) and capillary VAMS samples were collected at 2-, 6-,10-, 24-, 48- and 96-hours post-antibiotics administration. Antibiotic concentrations were determined using multiplex liquid chromatography coupled tandem mass spectrometry (LC-MS/MS). Statistical analyses included Mann-Whitney U tests and t-tests.

**Results:**

Significant differences in antibiotic concentrations were observed between VAMS and venous blood (VB) collection methods, across different time points for the three antibiotics (*p* < 0.05). AMO concentrations in VB were 3.5-fold higher (*p* < 0.01) than in VAMS at early time points (2, 6, 10 h (h)). MET levels in VB were 1.5-fold higher than in VAMS at 2 h and 6 h, (*p* < 0.01), but this difference disappeared after 10 h. Alternately, while AZI levels were similar in VB and VAMs 2 h after administration, AZI concentrations in VB and VAMS declined non parallelly, with VB levels decreasing to about 60 to 25% of those measured in VAMS over the observed 96 h interval. Antibiotic exposures were not different in the PH and PP groups. Differences in antibiotics concentrations determined in VB and VAMS samples are a direct consequence of (i) the matrices used for analyses (plasma in VB, vs. whole blood with VAMS), (ii) the subjects’ hematocrit, and (iii) the distinct cell distribution pattern of antibiotics with AMO characterized by a weak penetration in red blood cells (RBC) while AZI tends to progressively concentrate into RBC. MET was present at higher concentrations in plasma until 6 h which thereafter tended to re-equilibrate equally in plasma and RBC.

**Conclusion:**

Though VAMS yielded significantly different results compared to plasma, it effectively reflects the concentration evolution of the antibiotics and could be an alternative in pharmacokinetic studies and therapeutic monitoring.

**Clinical relevance:**

VAMS holds promise in advancing therapeutic drug monitoring in periodontal research and clinical practice. Being less invasive than venous puncture it is well accepted by subjects and facilitate blood monitoring in clinical trials and non-hospital settings. Its minimal invasiveness and simplified logistics make it suitable for enhancing precision medicine and pharmaceutical approaches in periodontology.

**Supplementary Information:**

The online version contains supplementary material available at 10.1007/s00784-025-06466-3.

## Introduction

The measurement of biochemical markers for health monitoring, disease diagnosis, and inflammatory markers identification, is generally performed with blood samples (~ 2–5 ml aliquots) collected by venipuncture and sent to centralized laboratories for centrifugation, and serum/plasma collection and storage [1]. However, analytical methods are becoming increasingly sensitive allowing a marked reduction in the required volume sample for analyses, particularly through microsampling approaches. Unlike the traditional venipuncture, which requires the collection of at least 1 mL of blood, microsampling typically requires 10 to 20 µL of blood and generally much less than 100 µL. One of the earliest methods, the dried blood spot (DBS) approach, is widely used for newborn screening of metabolic diseases [2].

In recent years, volumetric absorptive microsampling (VAMS) has emerged, offering the advantage of providing a fixed volume of collected blood, which improves analyte quantification regardless of hematocrit [3, 4]. VAMS involves a minimally invasive finger prick and allows for easy blood collection and storage without requiring venous access [5]. Importantly, this technique is well accepted by patients [6]. It is particularly suitable for blood collection outside hospital settings, where trained medical staff may not be available and, it simplifies sampling procedures in large-scale clinical studies.

The standardized 10 µL volume in VAMS offers significant advantages over DBS, particularly in terms of volume accuracy and hematocrit independence and pre-treatment. By ensuring a precise volume collection, VAMS reduces variability caused by hematocrit effects, leading to more accurate and reliable results [7].

However, a notable disadvantage of VAMS is its limitation in measuring concentration in whole blood, which can be problematic when plasma-based monitoring is required. Since certain analytes exhibit different concentrations in plasma compared to whole blood, direct comparisons or interpretations can be challenging. In such cases, additional steps or correction factors may be necessary to accurately correlate whole blood measurements with plasma concentrations [8].

VAMS has proven to be a valuable alternative tool in therapeutic drug monitoring (TDM) and for clinical pharmacokinetics studies [1]. According to the literature, VAMS has been used to identify inflammatory markers and holds potential in the field of dentistry, particularly periodontology, to study the association between periodontitis and various major systemic diseases such as atherosclerosis, diabetes, and adverse pregnancy outcomes [9]. These conditions are driven by bacteremia and systemic inflammation, with elevated inflammatory markers levels observed in periodontitis patients compared to healthy subjects [10, 11]. Identifying these markers in systemic circulation is both clinically and biologically relevant for investigating and addressing the bidirectional relationships among these diseases. The integration of VAMS could facilitate such analytical approaches.

Moreover, there is a growing interest in periodontology to evaluate the local and systemic distribution of antibiotics in patients undergoing dental treatment. The administration of systemic antibiotics in combination with non-surgical periodontal therapy, has shown significant clinical benefits, particularly for patients with deep periodontal pockets and aggressive periodontitis [12, 13]. Commonly used antibiotics include amoxicillin (with or without clavulanic acid), azithromycin, clindamycin, doxycycline, metronidazole, spiramycin, tetracycline, and their various combinations [14]. However, robust, evidence-based data remain insufficient to recommend a specific dosage regimen and, or optimal treatment duration for these agents. This knowledge gap primarily stems from limited data on antibiotic distribution in deep pockets, oral tissues and crevicular fluid [15], and on the optimal antibiotic concentrations required to improve clinical outcomes. Consequently, well-designed pharmacokinetic studies are necessary to optimize antibiotic selection and dosing regimens ensuring effective treatment of periodontitis, while enhancing clinical outcomes, minimizing toxicity, and reducing bacterial resistance emergence. For such investigations, the VAMS devices would offer a practical solution for dentists eliminating the need for traditional, invasive venous blood sampling.

Considering these clinical and research needs, the aim of our study was to compare two blood sampling methods for the simultaneous quantification of the three most used antibiotics in periodontal treatment: amoxicillin (AMO), metronidazole (MET), and azithromycin (AZI). These sampling approaches were evaluated in individuals with or without periodontitis, involving capillary blood collection by finger prick using VAMS, alongside parallel venous blood collection through the traditional venipuncture. Based on the distinct pharmacokinetic profiles of AMO, MET, and AZI—ranging from hydrophilic to lipophilic properties and varying capacities for cellular and tissue distribution—we hypothesized that concentrations obtained via plasma and VAMS would differ, particularly at early and late time points. Furthermore, as inflammation can alter vascular permeability and cellular uptake, we postulated that periodontitis might subtly affect antibiotic pharmacokinetics, especially for AZI, which is actively sequestered in immune cells and inflamed tissues. While this study does not directly measure antibiotic concentrations in periodontal pockets or tissues, it aims to evaluate whether VAMS, a minimally invasive method, can provide pharmacokinetic data comparable to plasma sampling. Establishing this equivalence is a critical step before designing studies that assess tissue-level antibiotic penetration using VAMS or other surrogate matrices.

## Materials and methods

### Ethical approval

This is a pilot, single-center, interventional, prospective clinical study. The protocol was approved by the Ethical Committee of the University Hospitals of Geneva, Geneva, Switzerland (N° 2022 − 00179) and was authorized by the Swiss Agency for Therapeutic Products (Swissmedic, Bern, Switzerland N° 701353). Research was conducted according to the principles outlined in the Declaration of Helsinki on experimentation involving human subjects. Written informed consent was provided by every patient before study inclusion and evaluation of recorded medical data. The study was supported by the Swiss National Science Foundation (Grant 32003B_212578/1) and funded partially by the SSP (Suisse Society of periodontology).

### Subjects

Subjects were recruited from the pool of patients seeking treatment in the Division of Regenerative Dental Medicine and Periodontology of the University Clinics of Dental Medicine between June 2022 and December 2022. Six periodontally healthy (PH) and six periodontally compromised volunteers aged between 29 and 56 years old were recruited. For inclusion, periodontitis patients (PP) had to present at least two teeth in the mouth with probing depth (PD) > 6 mm with bleeding on probing (BOP) and radiographic bone loss. Periodontally healthy participants (PH) presented no teeth with PD > 4 mm and had a total BOP score < 10%. The diagnosis and classification of periodontitis were performed according to the 2017 World Workshop on the Classification of Periodontal and Peri-Implant Diseases and Conditions, as described by Papapanou et al. [16]. Most participants in the PP group presented generalized Stage III, Grade B or C periodontitis, characterized by interdental clinical attachment loss ≥ 5 mm, probing depths > 6 mm, and radiographic bone loss extending to the middle third of root length or beyond. The pharmacokinetic measurements were performed prior to the initiation of subgingival instrumentation (i.e., before step 2 of periodontal therapy). To be included, participants should have no known contraindications (e.g. known hypersensitivity or allergy) to the study drugs AMO, AZI, MET, nor liver impairment or QT prolongation (extended QT interval on an electrocardiogram (ECG), which represents the time it takes for the heart’s ventricles to depolarize and repolarize. QT prolongation was evaluated based on existing medical records. No systematic ECG recordings were performed as part of the study protocol. This prolongation increases the risk of ventricular arrhythmias, particularly torsade de pointes (TdP)). Exclusion criteria comprise history of systemic diseases (cancer, HIV infection, bone metabolic diseases, radiation or immunosuppressive therapy), pregnancy or lactation, drug or alcohol abuse, and finally inability to understand and sign the informed consent form because of language problems or psychological disorders.

All adverse events were recorded throughout the study.

As this was a pilot study, no formal a priori power calculation was conducted. The sample size (*n* = 12) was based on logistical feasibility and the goal of estimating variability in VAMS and plasma measurements to inform future studies. The data generated herein will serve to refine effect size estimates for adequately powered confirmatory trials.

### Sample collection

Blood samples were collected in twelve volunteers (4 women and 8 men, aged 29–56 years old), at the Clinical Research Center of the University Hospital of Geneva (HUG). Each participant received *per os* the three antibiotics: 500 mg of AMO (Sandoz Pharmaceuticals AG, Switzerland), 500 mg of MET (Sanofi-Aventis SA, Switzerland), and 500 mg of AZI (Pfizer PFE GmbH, Switzerland). All three antibiotics were administered orally as a single combined dose (500 mg each) at the same time point (T0) for each participant. Paired venous whole blood and capillary blood (i.e. VAMS) samples were collected through devices (MITRA, Neoteryx, Torrance, CA, USA). VAMS samples were collected by trained healthcare professionals at the hospital service. Using an IV canula (intravenous cannula), venous blood sample (6mL) was collected on EDTA anticoagulant, at 2, 6 and 10, 24, 48, and 96 h after drug administration (T2, T6, T10, T24, T48, and T96, respectively). Following collection, the venous blood samples were centrifuged at 2000 g for 10 min at + 4 °C. The plasma was then separated and stored at −80 °C until analysis.

In parallel, a capillary blood sample was obtained by finger prick, and blood was collected using a VAMS 10 µL-device at the same time points (T2, T6, T10, T24, T48, and T96). The VAMS samples were prepared following the manufacturer’s instructions: the distal part of the tip was gently immersed in a drop of capillary blood, ensuring that the device was not fully submerged to prevent overfilling. After sample absorption, the tips were left to dry for two minutes before being placed on a dedicated rack and air-dried for 1 h at room temperature. The samples were stored at −80 °C in a sealed plastic box as per manufacturer recommendations.

AMO, MET, and AZI concentrations were quantified simultaneously in plasma and VAMS extracts using an in-house fully validated multiplex liquid chromatography coupled to tandem mass spectrometry (LC-MS/MS) methods. The process employed the respective stable isotopically labeled internal standards and matrix-matched calibration samples (i.e., blank plasma spiked with the analytes at several concentrations). All batches of blank plasma were obtained following institutional ethical standards from citrated blood collected from polycythemia vera patients undergoing routine phlebotomy at the Ambulatory Care Unit, Unisanté, University of Lausanne, Lausanne, Switzerland. Blood samples were centrifuged using a Hettich^®^ Rotanta 4600RF centrifuge at 2000 × g for 10 min at 4 °C.

Briefly, the sample (50 µL) underwent a protein precipitation with acentonitrol containg the internal standard. The chromatographic separations were performed using a Waters XSelect HSS T3 2.1 × 75 mm, 3.5 μm column (Waters^®^, Milford, MA, USA) in gradient mode, with a mobile phase composed of 0.2% formic acid and acetonitrile. AMO, MET, and AZI were detected by a triple-stage quadrupole mass spectrometer Quantis (Thermo Fischer Scientific, Waltham, MA, USA) equipped with an electrospray ionization (ESI) interface in positive mode. The Spray Voltage was set at 3500 V, a Capillary Temperature at 300 °C, and a Collision Gas Pressure of 1.5mTorr. The interval of quantification was 5–10’000 ng/ml for AMO and AZI, and 10–20’000 ng/ml for MET in both matrices. Method validation demonstrated intra- and inter-day coefficients of variation (CV) below 15%. Further details of the analytical method are provided in the Supplementary Material.

### Statistical analysis

Given the small sample size of this pilot study (*n* = 12), all analyses were performed using non-parametric statistical methods to minimize assumptions regarding data distribution. Paired comparisons between plasma and VAMS concentrations within the same individuals were conducted using the Wilcoxon signed-rank test. For group comparisons between patients with periodontitis (PP) and periodontally healthy individuals (PH), the Mann–Whitney U test was used. Statistical significance was set at *p* < 0.05. All analyses were conducted using, SPSS version 26.

## Results

A total of twelve subjects participated in and completed the study, with 6 being periodontally healthy and 6 having periodontitis. Table 1 presents the demographic information of the study participants. Seven subjects were systemically healthy, with no concomitant diseases. Among the PP group, one had type II diabetes managed through diet and exercise, another had hypertension, one had undergone thyroid removal, and another suffered from depression and prostatic hyperplasia. The cohort included 4 women and 8 men, with both sexes represented in each group. Additionally, 16.7% of the participants were smokers (2 out of 12) both in the PP group. The PH group was significantly younger, as younger individuals typically exhibit a lower prevalence of periodontal disease [17]. However, both groups were matched for all other criteria.

**Table 1:** Demographics of study participants in periodontally healthy (PH) and periodontally diseased individuals (PP)

Variables	PH (*N* = 6)	PP (*N*= 6)	Total (*N* = 12)	*P* value
Age (years)	33 ± 5	49± 5	41 ± 11	**0.024**
Height (cm)	175± 9	178± 9	177 ± 13	0.241
Weight (kg)	80± 20	92± 17	86 ± 17	1.000
BMI (kg/m^2^)	25.5± 13	28.9± 3	27.2± 3	0.336

Data are summarized as arithmetic mean ± standard deviation. *P* values were derived using the Mann-Whitney U test. Abbreviation: BMI, body mass index, p value: significant *p* < 0.05

**Fig. 1:**
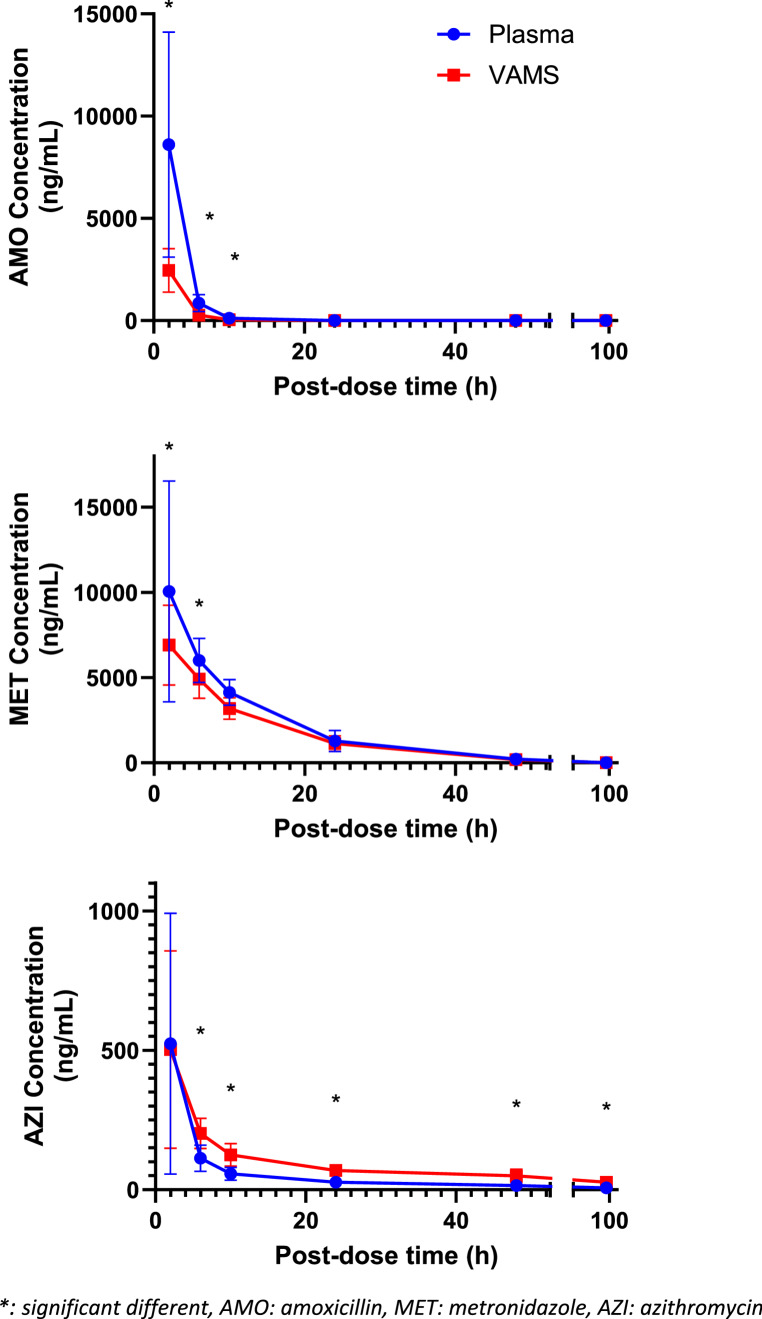
Mean antibiotic concentration (ng/mL) over time (hours) in all subjects (*n* = 12)

The mean blood concentration profiles of AMO, MET, and AZI at different time points, collected via both plasma and VAMS, are illustrated in detail in Table 2; Fig. 1. Significant differences in antibiotic concentrations were observed between volumetric absorptive microsampling (VAMS) and venous blood (VB) collection methods at various time points for the three antibiotics under study (*p* < 0.05). The magnitude and direction of these differences varied depending on the specific antibiotic and the time of measurement, reflecting distinct pharmacokinetic behaviors.

**Table 2:**
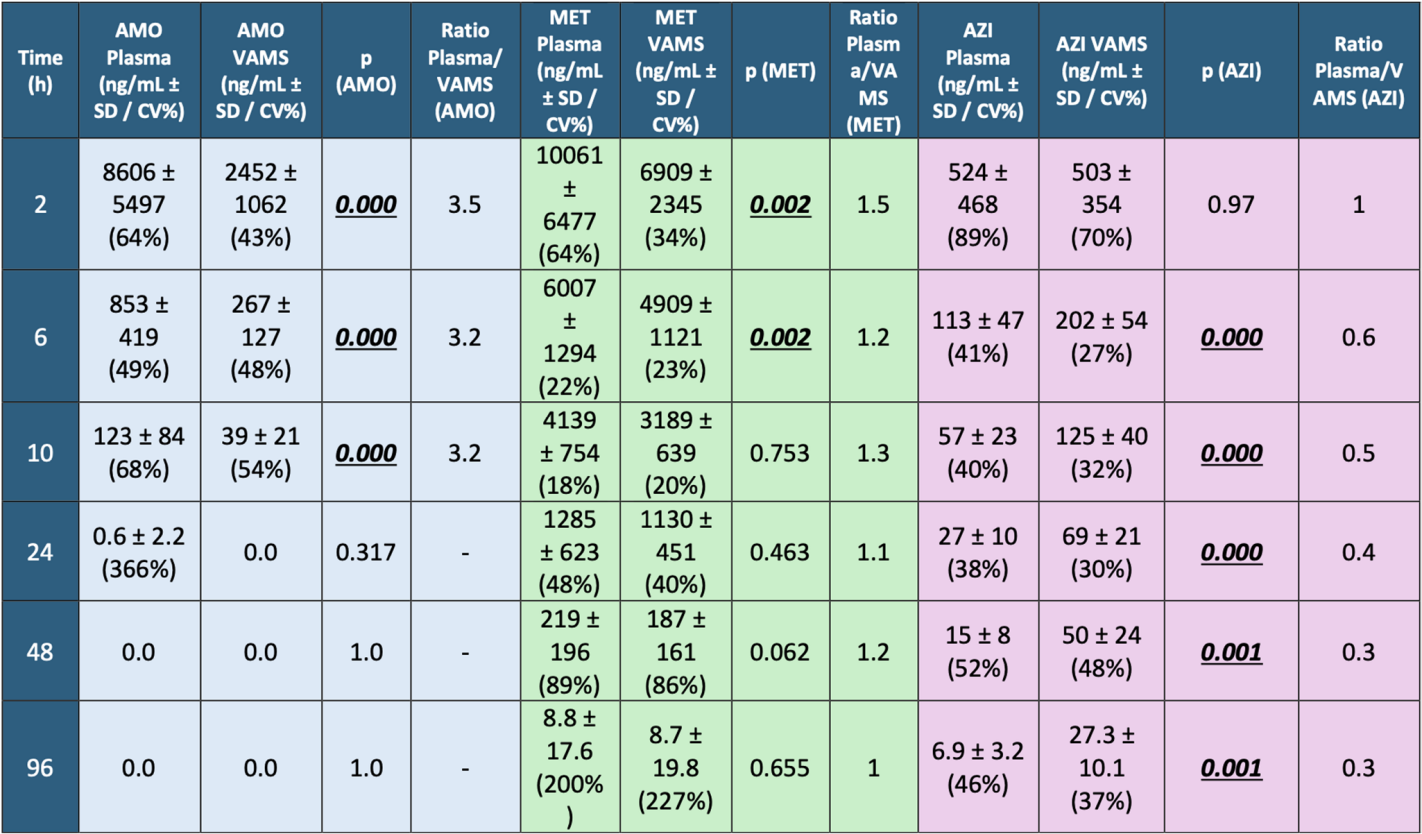
Mean (SD) antibiotic concentration (ng/mL) over time (hours) in all subjects (n=12)

*H: hours after antibiotic intake, AMO: amoxicillin, MET: metronidazole, AZI: azithromycin, p value: significant p<0.005*

For AMO, concentrations measured in VB were approximately 3.5-fold higher than those in VAMS at early time points (2-, 6-, and 10 h post-administration; *p* = 0.000 for each). At T2, the mean VB concentration was 8606 ± 5497 ng/mL (CV = 63.9%) compared to 2452 ± 1062 ng/mL (CV = 43.3%) in VAMS. This trend persisted at T6 and T10 but was longer statistically significant by 24 h (*p* = 0.317). AMO concentrations became undetectable in both VB and VAMS at T48 and T96. This pattern aligns with AMO’s known pharmacokinetic profile and suggests that VAMS can adequately track its clearance over time, even though initial concentrations may be underestimated.

For MET, significant differences in concentrations were observed at T2 and T6, with VB levels approximately 1.5-fold higher at T2 (*p* = 0.002), where VB concentrations were 10.061 ± 6477 ng/mL (CV = 64.3%) versus 6909 ± 2345 ng/mL (CV = 33.9%) in VAMS. At T6, VB levels remained significantly higher (6007 ± 1294 ng/mL, CV = 21.5%) compared to VAMS (4909 ± 1121 ng/mL, CV = 22.8%) with a VB/VAMS ratio of 1.2 (*p* = 0.002). However, at T10, concentrations equalize between the two matrices, and no statistically significant differences are observed at later points (*p* = 0.753, 0.463, 0.062, and 0.655 at 10, 24, 48, and 96 h, respectively). The observed convergence in concentrations after 6 h suggests that MET distributes more evenly between plasma and whole blood over time.

AZI displayed a distinct pattern compared to AMO and MET. While there was no significant difference at T2 (*p* = 0.97), VAMS concentrations remained consistently higher than VB from T6 onwards (*p* = 0.000 at 6, 10, 24, and *p* = 0.000 at 48 and 96 h), AZI concentrations measured via VAMS were consistently higher than those in VB. At T6, VB concentrations were 113 ± 47 ng/mL (CV = 41.4%) compared to 202 ± 54 ng/mL (CV = 26.7%) in VAMS, with a VB/VAMS ratio of 0.6. This pattern persisted, with VB values declining to approximately 40–30% of VAMS values at T48 and T96. For example, at T96, VB concentrations were 6.9 ± 3.2 ng/mL (CV = 46.4%) versus 27.3 ± 10.1 ng/mL (CV = 36.9%) in VAMS (*p* = 0.001, VB/VAMS ratio = 0.3). This pattern is consistent with AZI’s known intracellular accumulation and prolonged retention in red blood cells.

Additionally, no significant differences in the pharmacokinetic profiles of any antibiotics were observed between healthy individuals and those with periodontitis. This suggests that periodontitis does not significantly impact the absorption, distribution, or elimination of AMO, MET, or AZI. (Table 3). Most participants in the PP group presented generalized Stage III, Grade B or C periodontitis, characterized by interdental clinical attachment loss ≥ 5 mm, probing depths > 6 mm, and radiographic bone loss extending to the middle third of root length or beyond.

**Table 3:**
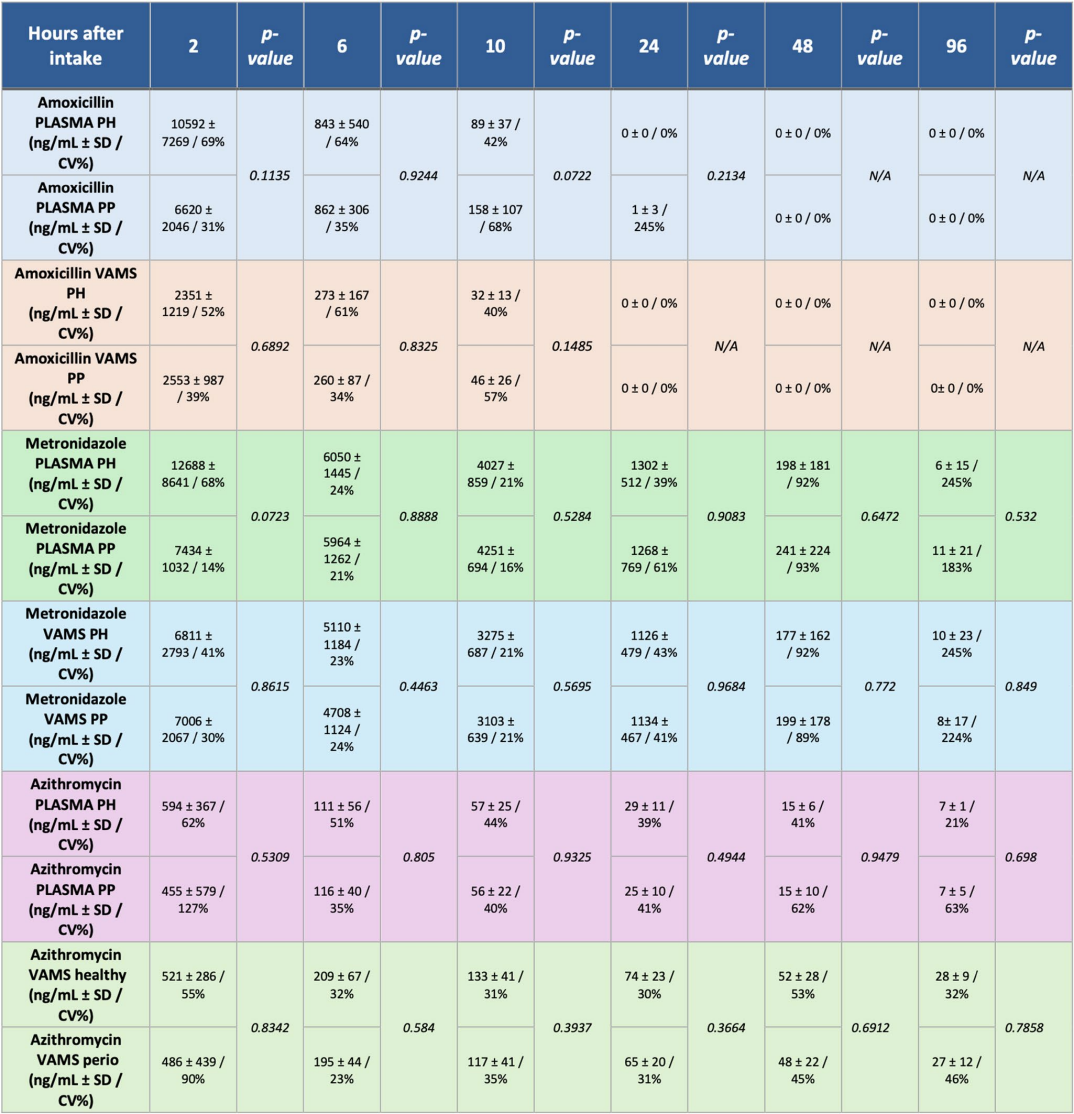
Mean antibiotic concentrations (ng/ml) over time (hours after intake) PH (n=6) vs PP (n=6)

*PH: participants without periodontal problems, PP: participants affected with periodontitis*

## Discussion

This study investigated the pharmacokinetics of AMO, MET, and AZI using both venous blood (VB) and volumetric absorptive microsampling (VAMS). VAMS has gained increasing attention in therapeutic drug monitoring (TDM) due to its minimally invasive nature and suitability for remote sampling. Our findings demonstrate that VAMS provides a reliable alternative for antibiotic pharmacokinetic assessments, albeit with some systematic differences compared to plasma-based venous blood sampling. The observed differences between VB and VAMS can be attributed to several factors, including analyte partitioning between plasma and red blood cells, matrix composition, and potential hematocrit effects. AMO, being primarily confined to plasma, exhibited higher concentrations in VB at early time points, whereas AZI, known for its intracellular accumulation, showed significantly higher concentrations in VAMS at later time points. MET displayed an intermediate profile, with early differences that later converged between the two matrices. These findings align with previous reports indicating that VAMS captures whole-blood drug levels, which may differ from plasma concentrations due to cell-associated drug distribution. This highlights the importance of considering the sampling matrix when interpreting pharmacokinetic data and the therapeutic range.

Despite these differences, VAMS accurately reflected the concentration evolution of all three antibiotics, demonstrating its potential as a viable alternative for pharmacokinetic studies, particularly in outpatient and non-hospital settings. The convenience and acceptability of VAMS, especially for patient-centric monitoring, underscore its potential for broader applications in clinical pharmacology and precision medicine.

Various studies have investigated the applicability of VAMS across diverse compounds, demonstrating its potential through proof-of-concept validations [18–24].

The VAMS method is also predominant in preclinical studies, especially in the early phase of drug development. Applications include a range of areas such as drug discovery, drug development, toxicokinetics, newborn screening, animal studies, clinical trials, therapeutic drug monitoring [25], metabolomics [26], proteinomics [27], and biomarkers monitoring [28]. The range of analytes is extended to various categories like antimicrobials and antibiotics [29, 30], paracetamol [31], anticancer agents [32], immunosuppressants, and antirheumatic drugs [5] and heavy metals [33].

According to a recent systematic review, there is increasing interest in using microsampling to generate and validate pharmacokinetic data, particularly to optimize antibiotic dosing. Studies typically compare data from conventional plasma samples with microsamples, revealing occasional discrepancies. The authors of the systematic review suggest that standardized validation tests specific to microsampling across biological fluids are essential to enhance confidence in clinical applications of VAMS [1].

In that scope, the present study aimed to compare the VB sampling and VAMS for determining the pharmacokinetic profiles of three antibiotics—AMO, MET and AZI. A total of 12 subjects, comprising 6 periodontally healthy individuals and 6 subjects with periodontitis, were enrolled. The study subjects were evenly matched in demographic characteristics, showing no notable disparities in height, weight, or BMI between those classified as periodontally healthy and those with periodontal disease. Participants in the PH group were slightly younger, which can be attributed to the higher prevalence of periodontal disease in older individuals. Most subjects were systemically healthy, although some periodontal patients had conditions, which could potentially influence drug metabolism and pharmacokinetics. However, the presence of these conditions did not significantly impact the overall pharmacokinetic profiles of the antibiotics studied, as indicated by the lack of significant differences between the healthy and periodontitis groups. Most participants in the PP group presented generalized Stage III, Grade B or C periodontitis, characterized by interdental clinical attachment loss ≥ 5 mm, probing depths > 6 mm, and radiographic bone loss extending to the middle third of root length or beyond. Despite this moderate to severe disease profile, the small sample size likely limited the statistical power to detect significant intergroup differences.

Our results show distinct pharmacokinetic patterns for each antibiotic depending on the method of blood collection and provide insights into the applicability of VAMS in clinical settings. The choice between whole blood and plasma sampling can significantly influence the pharmacokinetic profiling of antibiotics such as AMO, MET and AZI. Whole blood contains all cellular components, including red blood cells, white blood cells, and platelets, in addition to plasma, while plasma is the acellular fluid obtained by centrifuging whole blood collected with anticoagulants. The distribution of antibiotics between plasma and blood cells can substantially affect the measured drug concentrations. For instance, lipophilic antibiotics like AZI (log *P* ~ 3) [34]may distribute into red blood cells, leading to higher concentrations in whole blood compared to plasma. In contrast, more hydrophilic antibiotics like AMO (log *P* ~ 0.7) [34]and even MET (log *P* ~ 1.7) [34], are more distributed in the aqueous plasma phase, resulting in more consistent concentration measurements between the two matrices [35–37].

More specifically in our study, AMO exhibited higher plasma concentrations at all time points when measured via VB compared to VAMS. This trend aligns with the known distribution of amoxicillin, which is primarily restricted to plasma due to its limited penetration into red blood cells [38].

MET showed comparable concentration profiles between VB and VAMS at most time points. However, significant differences were observed at 2 and 6 h (*p* < 0.005), with notably higher plasma concentrations measured in VB. This observation may be attributed to differences in the diffusion of MET across red blood cells, as MET is known to partition more evenly between plasma and whole blood over time.

AZI displayed higher concentrations in VAMS samples at later time points compared to VB, suggesting slower elimination or prolonged presence when measured via VAMS. This may be linked to AZI affinity for intracellular compartments, particularly red blood cells [39]. AZI, in contrast to MET and AMO, has also a large volume of distribution and a long half-life. It is actively taken up by neutrophils, macrophages, and fibroblasts, especially in inflamed tissues, including gingiva [39, 40]. This accumulation explains its sustained concentrations in peripheral compartments and highlights the relevance of whole-blood sampling when evaluating AZI pharmacokinetics. The higher concentrations detected in VAMS may be related to the whole blood sampling approach of VAMS, which captures intracellular drug content. Similar findings were reported by Denniff et al., who observed higher maximum concentrations (Cmax) for paracetamol using VAMS in comparison to conventional EDTA-tube collection in rats. Given its log P of 0.5, this difference is unlikely to be related to its hydrophilic-lipophilic balance [41]. Such evidence supports the ability of VAMS to capture a more holistic view of drug distribution.

The type of sampling matrix could also influence analyte recovery. Similar findings to ours were reported by Takyi-Williams et al., who assessed the simultaneous quantification of six antibiotics using VAMS. Their study demonstrated reproducible analyte recovery, with recovery rates around 45% for vancomycin and over 80% for other antibiotics, including ampicillin, cefepime, ceftriaxone, meropenem, piperacillin, and tazobactam [29]. This illustrates how VAMS’ performance may vary depending on the physicochemical properties of the analyte.

One limitation of this study lies in the lack of direct validation of matrix-related effects. The observed discrepancies between VAMS and plasma concentrations may result from physiological factors such as the differential distribution of antibiotics between plasma and cellular compartments, as well as analytical factors including matrix effects during extraction and variability in recovery efficiency. Performing recovery validation using spiked control samples prepared in both plasma and whole blood matrices would have enabled a more accurate evaluation of these inter-matrix differences. Without this comparison, it remains unclear whether the observed differences reflect true biological variability or are partly due to methodological bias. This step was not included in the present study but will be addressed in future validation protocols.

To sum up, it is important to consider the method of blood collection when interpreting pharmacokinetic data for these antibiotics. VAMS proved comparable to venous blood collection, in tracking the concentration decrease for pharmacokinetic studies of antibiotics in periodontal therapy. The application of VAMS in this study is in accordance with findings from various other research efforts that have validated the use of VAMS for different drugs and biological matrices. For instance, Veenhof et al. demonstrated that VAMS and dried blood spots (DBS) could reliably monitor tacrolimus levels in kidney transplant patients, although DBS showed slightly better performance [42]. Studies such as those by Breken et al. [43], which analyzed clozapine concentrations, and Marasca et al. 2020, which investigated antidepressants in saliva, further reinforce the adaptability and accuracy of VAMS for therapeutic drug monitoring in diverse clinical settings [44].

## Conclusion

This pilot study highlights the potential of VAMS as a minimally invasive and effective tool for antibiotics pharmacokinetic assessments in periodontal treatment. While significant differences in antibiotic concentrations were observed between VAMS and traditional venous blood sampling, VAMS successfully tracked concentration changes over time. The overall pharmacokinetic trends were comparable, demonstrating the feasibility of VAMS for therapeutic drug monitoring.

Given its ease of use, patient acceptability, and suitability for remote sampling, VAMS holds promise for expanding pharmacokinetic research and clinical drug monitoring beyond traditional hospital settings.

Future studies with larger sample sizes and a broader range of antibiotics are needed to further validate these findings and optimize VAMS applications in routine practice and in personalized medicine.

## Supplementary Information

Below is the link to the electronic supplementary material.

Supplementary Material 1

## Data Availability

No datasets were generated or analysed during the current study.
