# Supplementary material for: A comparison between venous blood sampling and capillary volumetric absorptive microsampling for antibiotics levels monitoring in individuals with and without periodontal disease
Source: Clin Oral Investig. 2025 Aug 23;29(9):420. doi: 10.1007/s00784-025-06466-3 (PMC12374872; doi:10.1007/s00784-025-06466-3)

**Supplementary material - Materials and Methods**

**Sample Preparation**

1. Thaw all biological samples and calibration standards stored at −80°C.
2. Pre-cool the centrifuge to +4°C by running it empty.
3. Transfer 50 µL of each sample into a 1.5 mL Eppendorf tube.
4. Add 150 µL of internal standard (IS) solution to each tube (final concentrations of IS, i.e. Amoxicillin-d4, Metronidazole-d4 (MET-d4), Azithromycin-d5 ): 200 ng/mL)
5. Vortex each tube for 5 seconds.
6. Sonicate for 30 seconds to promote protein precipitation.
7. Centrifuge the tubes at 12,700 rpm for 10 minutes at 4°C.
8. Transfer 150 µL of the resulting supernatant to an HPLC vial.
9. Add 150 µL of ultrapure water (H₂O) to the vial and seal using HPLC-compatible blue pressure caps.
10. Vortex briefly to ensure complete homogenization of the solution.

**HPLC Conditions**

| **Time (min)** | **Solvent A (%)** | **Solvent B (%)** | **Flow Rate (µL/min)** |
| --- | --- | --- | --- |
| 0.00 | 90 | 10 | 300 |
| 3.00 | 55 | 45 | 300 |
| 5.00 | 10 | 90 | 300 |
| 5.10 | 90 | 10 | 300 |
| 7.00 | 90 | 10 | 300 |

- **Solvent A**: 0.2% formic acid in water
- **Solvent B**: Acetonitrile
- **Column**: Waters XSelect HSS T3 2.1 x 75 mm, 3.5 µm column (Waters^®^, Milford, MA, USA)

**LC-MS/MS Parameters**

- Spray Voltage: 3500 V
- Sheath Gas Pressure: 50 arbitrary units
- Auxiliary Gas Pressure: 15 arbitrary units
- Capillary Temperature: 300°C
- Q2 Collision Gas Pressure: 1.5 mTorr
- Q1 and Q3 Peak Width: 1.20 FWHM (Full Width at Half Maximum)
- Chromatographic Peak Width: 12 s
- Cycle Time: 0.2 s

**MRM Transitions and Retention Times**

| **Compound** | **Retention Time (min)** | **Transition (m/z)** | **Collision Energy (eV)** |
| --- | --- | --- | --- |
| Metronidazole | 1.6 | 172.1 → 128.0 | 14 |
| Metronidazole-d4 | 1.6 | 176.1 → 128.0 | 13 |
| Azithromycin | 2.6 | 749.6 → 591.4 | 28 |
| Azithromycin-d5 | 2.6 | 754.6 → 596.4 | 29 |
| Amoxicillin | 3.3 | 366.1 → 349.1 | 7 |
| Amoxicillin-d5 | 3.3 | 371.1 → 354.1 | 7 |

**Validation of the method**

| **Compound** | **Quantification range (ng/ml)** | **CV intra-day (%)** | **CV inter-day (%)** |
| --- | --- | --- | --- |
| Metronidazole | 10- 20’000 | 3.1 - 3.8% | 3.1 - 3.8% |
| Azithromycin | 5-10’000 | 3.3 - 4.6% | 3.3 - 4.6% |
| Amoxicillin | 5-10’000 | 3.4 - 3.9% | 3.4 - 3.9% |

**Representative Chromatogram**


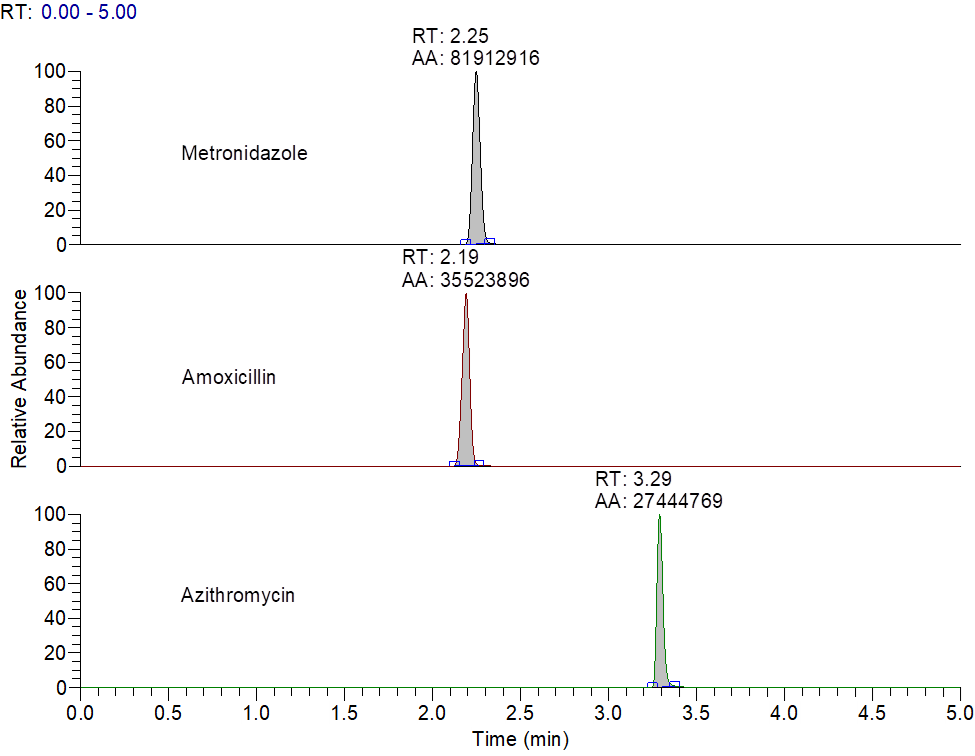

Supplement: Supplementary file 1 — Supplementary Material 1 [file 784_2025_6466_MOESM1_ESM.docx]
